# Comparative Study of Different Additive Manufacturing Methods for H13 Tool Steel

**DOI:** 10.3390/ma18235299

**Published:** 2025-11-24

**Authors:** Paweł Widomski, Marcin Kaszuba, Daniel Dobras, Dominik Terefinko, Michał Kołodziński

**Affiliations:** 1 Department of Metal Forming, Welding and Metrology, Faculty of Mechanical Engineering, Wroclaw University of Science and Technology, 50-371 Wrocław, Poland; marcin.kaszuba@pwr.edu.pl (M.K.); daniel.dobras@pwr.edu.pl (D.D.); michal.kolodzinski@pwr.edu.pl (M.K.); 2 Department of Analytical Chemistry and Chemical Metallurgy, Faculty of Chemistry, Wroclaw University of Science and Technology, 50-371 Wrocław, Poland

**Keywords:** additive manufacturing, H13 tool steel, Fused Deposition Modeling and Sintering (FDMS), laser powder bed fusion (LPBF), directed energy deposition (DED), microstructure, porosity, heat treatment

## Abstract

Additive manufacturing (AM) of hot-work tool steels such as H13 offers unique opportunities for producing complex, conformally cooled tools with reduced production time and material waste. In this study, five metal AM technologies—Fused Deposition Modeling and Sintering (FDMS, Desktop Metal Studio System and Zetamix), Binder Jetting (BJ), Laser Powder Bed Fusion (LPBF), and Directed Energy Deposition (DED)—were compared in terms of microstructure, porosity, and post-processing heat treatment response. The as-printed microstructures revealed distinct differences among the technologies: FDMS and BJ exhibited high porosity (6–9%), whereas LPBF and DED achieved near-full densification (<0.1%). Samples with sufficiently low porosity (BJ, LPBF, DED) were subjected to tempering and quenching treatments to evaluate hardness evolution and microstructural transformations. The satisfactory post-treatment hardness was observed in both tempered and quenched and tempered BJ samples, associated with secondary carbide precipitation, while LPBF and DED samples retained stable martensitic structures with hardness around 600 HV0.5. Microstructural analyses confirmed the dependence of phase morphology and carbide distribution on the thermal history intrinsic to each AM process. The study demonstrates that while FDMS and BJ are more accessible and cost-effective for low-density prototypes, LPBF and DED offer superior density and mechanical integrity suitable for functional tooling applications.

## 1. Introduction

Tools’ wear resistance and suitability for high-temperature service are due to the properties of hot-work tool steels. Usually, using conventional steels such as X37CrMoV5-1 (WCL) or X40CrMoV5-1 (WCLV) provides sufficient strength for hot forging. However, such steels offer a service life on the order of a few hundred to a few thousand forgings produced with a single tool; therefore, for serial production, it is recommended to introduce improvements to the tool material, such as selecting a more resistant grade or modifying the heat treatment used.

At present, hot-work tool steels with increased strength, high hardness, and, at the same time, adequate resistance to brittle fracture dominate. The basic, commonly used grades include low-alloy steels intended for hammer forging, such as 55NiCrMoV7 (WNL) and 35CrMo8 (WNLV), as well as steels for press forging, such as X37CrMoV5-1 (WCL, H11), X38CrMoV5-3 (1.2367), X40CrMoV5-1 (WCLV, H13), X32CrMoV33 (WLV), etc.

As a result of advances in metallurgy, many steels with enhanced mechanical properties and a greater ability to operate at high temperatures have been introduced to the market. Examples include products from Uddeholm-Böhler (Voestalpine) and Schmolz + Bickenbach, such as the Unimax, Dievar, and Hotvar grades, or the Thermodur series. For example, Unimax steel exhibits markedly higher tempering resistance at elevated temperatures and a multiple-times longer service life [1]. It has also been demonstrated that, at comparable hardness, these steels are more resistant to brittle fracture.

Research is still ongoing into the development of new grades of hot-work steels, particularly with respect to modifying their chemical composition [2,3]. Another way to improve a tool material is to increase its toughness—that is, to reduce its brittleness. The main causes of brittle fracture are discontinuities present in steels and the segregation of alloying elements. The development of new steel remelting methods has largely contributed to achieving greater homogeneity and chemical purity [4]. It has been demonstrated that applying electroslag remelting (ESR) and homogenizing annealing has a positive effect on the extent of segregation of the elements most critical to the properties of the tool steels analyzed: C, Cr, Mo, and V [5]. Numerous studies confirm that electroslag-remelted steel is characterized by a finer and more uniform grain, and the material is more homogeneous and contains fewer defects and impurities [6]. The next stage of technological progress in steel remelting is the use of ESR with the addition of Ca to bind residual aluminum oxides (calcium treatment), combined with homogenizing annealing. Applying such a complex treatment has a positive effect on the fatigue life of tool steels.

The impact of heat treatment on the durability of forging tools is enormous. Errors made at this stage are the most common cause of tools being withdrawn from service prematurely [7]. Hardness—which provides resistance to abrasive wear—depends on heat treatment. Heat treatment also affects fatigue life, stemming from the course of austenitizing, the efficiency of the martensitic transformation, and, among other factors, the morphology of carbide precipitates of alloying elements. Heat treatment can be improved by optimizing the time parameters and temperature gradients during individual operations, which results in a much longer service life and durability of tools [8].

To increase the fatigue life of forging tools, decarburization and oxidation of their surfaces during heat treatment should be avoided. Sometimes, to prevent surface decarburization and oxidation, heat treatment is carried out in furnaces with a protective atmosphere. This is particularly useful for surfaces that are not machined after heat treatment [9].

An effective method that has received little attention in the literature due to its limited popularity is subzero and cryogenic treatment (long-term deep freezing), which also serves to increase the service life of hot-work steels [10]. Subzero treatment consists of cooling quenched steel to below zero (approx. −70 °C) and holding it there to more fully complete the martensitic transformation and eliminate excess retained austenite from the steel’s microstructure. Subzero treatment improves the material’s mechanical and fatigue strength and enhances the dimensional stability of tools produced in this way [11]. Cryogenic treatment (long-term deep freezing) involves cooling the steel to about −180 °C and holding it there for a specified time. In deep cryogenic treatment, retained austenite is eliminated and more carbide precipitates form, which leads to improved wear resistance of the tools [12,13].

Understanding the transformation kinetics of tool steels during heat treatment is fundamental to optimizing their service performance. Comprehensive studies on high-alloy tool steels—including investigations of quenching and tempering effects on tribological properties of premium powder grades such as ASP2017 and ASP2055 compared against X153CrMoV12—have demonstrated the critical relationship between heat treatment parameters and hardness development [14]. Furthermore, detailed continuous cooling transformation (CCT) diagrams established through dilatometric analysis of materials such as X153CrMoV12, 100MnCrW4, and M398 powder metallurgy tool steel have clarified the microstructural mechanisms underlying martensite formation and secondary carbide precipitation, providing essential reference data for interpreting the thermal response of additively manufactured tool steel components [15,16].

Hot-work tool steels such as AISI H13 (X40CrMoV5-1) underpin dies and molds for high-temperature, high-pressure operations—die casting, hot extrusion, and forging—thanks to their balance of hot strength, temper resistance, toughness, and wear resistance. Additive manufacturing (AM) broadens that value proposition by enabling conformal cooling, graded structures, and repair, which can reduce cycle times and extend tool life relative to conventionally machined inserts. Landmark demonstrations of conformally cooled H13 hardware and subsequent thermo-mechanical analyses established both the design latitude of AM and the performance benefits specific to H13 [17,18].

Alongside conventionally used technologies, recent years have seen the emergence of new methods for producing tool steels such as AISI H13 based on additive manufacturing—that is, 3D printing. Although they do not match the productivity of conventional methods, they offer new possibilities for multimaterial structures and the creation of internal cooling channels, and are therefore seeing increasing use in the tooling industry.

Within the AM process space, laser powder bed fusion (LPBF/SLM) has been the most widely explored route for H13. Dense builds (~99–99.9%) and robust hardness after tempering were shown for SLM H13, while sensitivity to process parameters and heat treatment was mapped in detail [19,20,21]. At the same time, residual-stress and cracking challenges—arising from steep thermal gradients and H13’s hardenability—were quantified, with mitigation via preheating and tailored scan strategies [22,23,24,25]. Recent work has further pushed strength–toughness combinations, reporting ~2 GPa tensile strengths after appropriate tempering of LPBF H13 [26].

Directed-energy processes complement PBF for H13. Laser directed energy deposition (laser cladding) has long been used for near-net builds and repair of H13, offering high deposition rates and metallurgical bonding; studies document tempered-martensitic overlays with competitive hardness and wear [27,28]. Wire-arc additive manufacturing (WAAM) with H13 wire enables centimeter-scale walls and large tools at low cost per volume; representative investigations span microstructure control, elevated-temperature properties, and build-strategy effects [29,30,31,32].

Beyond melt-based routes, binder-assisted processes are increasingly attractive for H13 because they decouple shaping from densification. Binder-jetting of H13 followed by supersolidus liquid-phase sintering (SLPS) has achieved nearly full densification and clarified process windows and microstructure–property relations, making the approach relevant for series production of complex tools [33,34].

A closely related family—material extrusion of metal feedstocks (often termed bound-metal extrusion, fused deposition of metals, or FDMS/MEX) is especially compelling for shop-floor adoption: hardware is inexpensive, handling is safe (powders bound in a polymer), and toolpaths mirror established FFF workflows. Historically validated on stainless and Ni-based systems, MEX has very recently matured for H13 as well. A complete, cost-oriented route from extrusion printing through debinding and sintering has been demonstrated specifically for H13, with mechanical properties improved through post-processing [35]. Independent studies on MEX-H13 report tensile strengths approaching ~1.2 GPa in favorable build orientations and practical machinability after sintering—critical for finishing inserts—while also documenting porosity and surface-quality limits that motivate HIP/heat-treatment and toolpath optimization [36,37]. Taken together, these data position extrusion-based AM as the simplest and lowest-cost entry point to printed H13 tooling, now with evidence not only across other alloys but increasingly for H13 itself.

Despite extensive literature review, a comprehensive comparison of H13 tool steel manufactured using different additive manufacturing techniques (LPBF, DED, EBM, BJ, FDMS) has not yet been published. Comparative studies across multiple AM techniques remain limited, even for materials structurally similar to H13. However, relevant multi-method comparative studies exist for analogous materials: 316L stainless steel has been compared across LPBF, DED, and other techniques, demonstrating significant differences in mechanical properties, microstructure, and density [38]. Titanium alloys (i.e., Ti-6Al-4V) have been systematically investigated through multi-method comparisons encompassing LPBF, EBM, and DED, analyzing dimensional accuracy, mechanical properties, and microstructural characteristics [39]. Recent comparative analyses of Fe-based alloys produced by DED and LPBF have provided frameworks for understanding process-property relationships that could be adapted for H13 steel [40,41]. Although these dispersed investigations on comparable materials suggest that each AM technique imparts unique microstructural and mechanical characteristics, a systematic comparison of all major AM methods applied to H13 steel under unified experimental conditions remains absent, representing a critical research gap in tool steel additive manufacturing literature.

In this study, an attempt was made to qualitatively compare printing with these methods using the popular H13 steel as an example, as it is dedicated to manufacturing tools for forging processes. The following methods were tested: Fused Deposition Modeling and Sintering (FDMS) in two technologies from Desktop Metal and Zetamix, Binder Jetting (BJ), LPBF and Directed Energy Deposition (DED) method—Wire Laser Metal Deposition (Wire LMD). The printed parts were expected to meet quality criteria such as low porosity, metallurgical cleanliness, uniformity of structure and properties, a hardness of at least 500 HV, and a tempered martensite microstructure after heat treatment.

## 2. Materials and Methods

The results include microstructure and porosity examinations of as-printed samples for five different manufacturing technologies: FDMS (Desktop Metal Studio System), FDMS (Zetamix), Binder Jetting, LPBF, and DED. Due to the high porosity of the steel produced by the FDMS method, these samples were omitted from further studies on the effect of heat treatment on the properties and structure of the printed steel. Those studies comprised microhardness measurements and microstructure analysis.

### 2.1. Materials

AISI H13 (DIN 1.2344, JIS SKD61, ISO X40CrMoV5-1) is a chromium-molybdenum hot work tool steel widely employed in demanding applications requiring resistance to thermal fatigue, wear, and high-temperature degradation. The material is available in multiple forms: as forged ingots for conventional machining, as atomized powder for laser powder bed fusion (LPBF) and powder metallurgy applications, as binder jetting feedstock, and as wire feedstock for directed energy deposition (DED). The nominal chemical composition conforms to AISI H13 specifications with the following typical alloying elements (Table 1), providing the exceptional combination of hardness and toughness that characterizes this steel grade. 

For this investigation, H13 steel was processed via five distinct additive manufacturing routes. Powder morphology and size distribution were selected or optimized for each technology to maximize densification and processing stability, as detailed in Table 2. The LPBF feedstock comprised spherical, gas-atomized H13 powder conforming to commercial specifications. The FDMS (Fused Deposition of Metals) powder was extracted from commercial H13 filament, yielding a bimodal size distribution (dv50 = 18.4 ± 1.6 μm, range dv10 = 9.5–44 μm). Binder jetting powder was sourced from a commercial supplier with particle size optimized for binder infiltration in the 20–45 μm range. DED utilized H13 wire feedstock with composition conforming to AISI H13 standards. Full characterization of powder chemistry and morphology appears in Table 1 and Table 2, respectively.

### 2.2. Parameters of AM Methods

For each method, optimal parameters were adopted, based on the manufacturers’ recommendations and our own preliminary studies.

#### 2.2.1. FDMS—Desktop Metal Studio System

This method uses a composite material containing H13 tool steel powder, a thermoplastic binder, and technical wax, intended for 3D printing using the FDMS technology. The manufacturing process comprises three key stages: 3D printing, debinding (binder removal), and sintering.

3D printing parameters: extrusion temperature 150 °C, bed temperature 25 °C, nozzle diameter 0.4 mm, layer height 0.20 mm, print speed 10 mm/s, no retraction.

Debinding parameters: a two-step process including chemical debinding in acetone (42 °C, 48 h, mass loss 5–6%) and thermal debinding (20–600 °C) in an Ar/H_2_ atmosphere (97.5/2.5).

Sintering parameters: a three-stage process involving presintering at 600–900 °C; intermediate sintering at 900–1100 °C, during which porosity drops to about 8%; and final sintering at 1100–1400 °C to obtain the finished part. Sintering shrinkage is 15–20% in the X, Y, and Z directions.

#### 2.2.2. FDMS—Zetamix

Zetamix H13 filament is a composite material containing 90 wt.% H13 tool steel powder (particle size 5–20 μm) and 10 wt.% polyolefin-based thermoplastic binder, intended for 3D printing using FDMS technology. The manufacturing process comprises three key stages: 3D printing, debinding (binder removal), and sintering.

3D printing parameters: extrusion temperature 180 °C, bed temperature 25 °C, nozzle diameter 0.6 mm, layer height 0.20 mm, print speed 10 mm/s, no retraction, fan 100%, minimum of 3 perimeter walls.

Debinding parameters: single-stage process involving thermal debinding (50–650 °C, heating rate 10 °C/h) in an Ar/H_2_ atmosphere (97.5/2.5).

Sintering parameters: temperature 1350 °C, heating rate 50 °C/h, soak 2 h, Ar/H_2_ (97.5/2.5) atmosphere, pressure 0.2 bar, gas flow 0.5 L/min. Sintering shrinkage is 15.4 ± 1% (X, Y) and 16.4 ± 1% (Z).

#### 2.2.3. Binder Jetting

Binder Jetting—on a Desktop Metal (formerly ExOne) system—the base material is H13 steel powder with a particle size of 22 µm, which is bonded using a special binder composed of PEG polymers and an alcohol solvent. The manufacturing process comprises three key stages: 3D printing, debinding (binder removal), and sintering.

3D printing parameters: layer thickness 70 µm, binder saturation 80%, binder drying temperature 35 °C, layer drying time 10–30 s depending on part geometry.

Debinding parameters: single-stage process involving thermal debinding (500–800 °C).

Sintering parameters: temperature 1310 °C, heating rate 50 °C/h, hold 4 h, under argon atmosphere. Sintering shrinkage is 16 ± 2% in the X, Y, and Z directions.

#### 2.2.4. LPBF

Samples produced using the LPBF method were fabricated on an Aconity Mini device (Aconity3D GmbH, Herzogenrath, Germany) at the Multidisciplinary Research Centre (Dziekanów Leśny, Józefosław, Poland). The base material was H13 steel powder with a particle size of 45 µm and moisture content below 0.1%, ensuring a stable manufacturing process.

3D printing parameters: layer thickness 60 µm, laser power 250 W, scanning speed 1050 mm/s, powder temperature 350 °C, hatch distance 70 µm.

#### 2.2.5. DED

Samples produced using the DED (Wire LMD) method were fabricated on a Meltio M450 system (Linares, Spain). The base material was H13 steel welding wire with a diameter of 1 mm, melted using six diode lasers with a total power of 200 W each.

3D printing parameters: layer thickness 0.8 mm, laser power 1200 W, printing speed 350 mm/min, shielding gas flow rate 10,000 mL/h (argon), bead overlap 10%

### 2.3. Methods for Testing and Characterization in “As-Printed” State

The specimen for the Light Optical Microscopy (LOM) analysis was cut parallel to the printing direction (perpendicular to the layers) and etched with 2% nitric acid reagent and 98% ethanol. The microstructure was observed using an Olympus GX51 optical microscope (Tokyo, Japan). A VEGA3 TESCAN scanning electron microscope (Brno, Czech Republic) working in secondary electron (SE) mode at 30 kV was used to determine the elemental distribution through the Energy Dispersive Spectroscopy (EDS).

### 2.4. Methodology of Evaluation of Heat Treatment Influence on Microstructure and Properties of Printed H13 Steel

**Figure 1:**
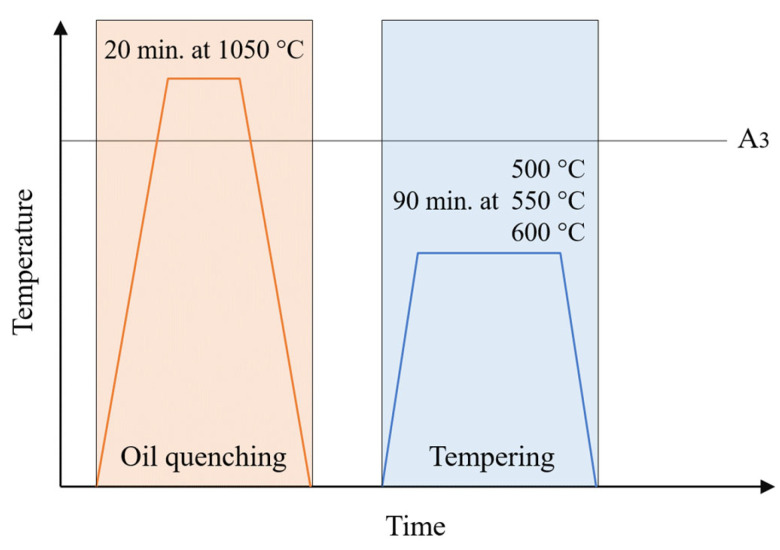
Heat treatment process diagram.

The samples that exhibited low porosity directly after printing by a given method (Binder Jetting, LPBF, and DED) were subjected to additional post-processing heat treatment—tempering (T), quenching (Q), or quenching and tempering (Q & T). Quenching was carried out in oil after holding the sample for 20 min at 1050 °C. Each tempering process lasted 90 min. The samples were cooled freely together with the furnace. A schematic of the heat treatment process is shown below in Figure 1.

For each of the three mentioned H13 steel fabrication methods (Binder Jetting, LPBF, and DED), the heat treatment was conducted in the following variants:

- Tempering at 500 °C
- Tempering at 550 °C
- Tempering at 600 °C
- Quenching
- Quenching + tempering at 500 °C
- Quenching + tempering at 550 °C
- Quenching + tempering at 600 °C

The choice of different tempering temperatures was motivated by the varied expectations associated with H13 steel. Specifically, the North American Die Casting Association (NADCA) standard #207 specifies the tempering requirements for H13 steel at 565 °C for the first two tempers and 550 °C for the third temper NADCA #207-2022 [43]. AISI standards recommend a tempering range between 538 °C and 649 °C [44]. The European DIN standard (1.2344) specifies a tempering range of 550–650 °C, while steel manufacturers provide varying guidelines depending on specific applications. Therefore, the authors selected three tempering temperatures (500, 550, and 600 °C), acknowledging that this range reveals the suitability of the printed steel for various applications.

The Vickers microhardness was measured by the LECO LM100AT hardness tester under the load of 500 g (St. Joseph, MI, USA). Six hardness measurements were made for each sample, inside the grain. The presented result is the average of the measurements. The ImageJ software (v1.x) was used to evaluate the porosity of the as-printed samples in the unetched state. A minimum pore area of 10 µm^2^ was applied. The microstructure of the samples was observed using the same microscope as in Section 2.3.

## 3. Results

The results include microstructure and porosity analyses for all technologies examined in the as-printed condition (Section 3.1 and Section 3.2), as well as the results of studies on the effect of heat treatment on hardness and microstructure for three selected technologies that exhibited satisfactorily low porosity (Section 3.3 and Section 3.4).

### 3.1. Microstructure in “As—Printed” State

A preliminary analysis of the microstructure of the samples in the as-printed condition was carried out. These analyses were performed for five samples produced using different variants of additive manufacturing technologies (two types of FDMS (Desktop Metal Studio System and Zetamix), BJ, LPBF, and DED).

**Figure 2:**
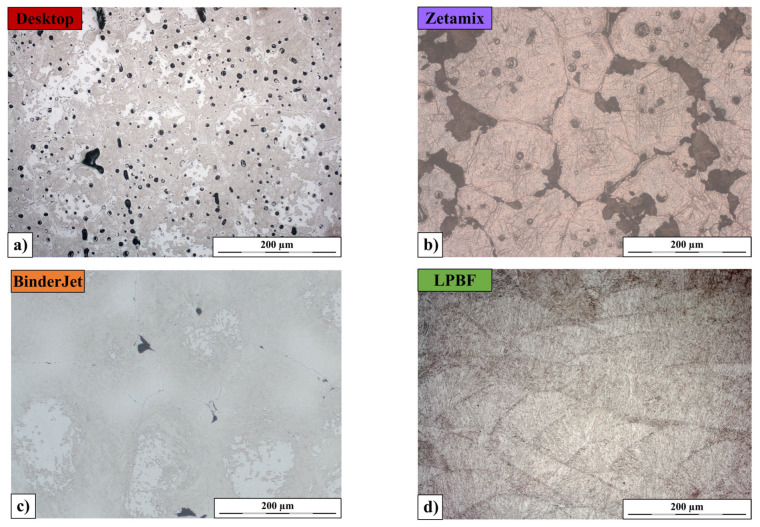

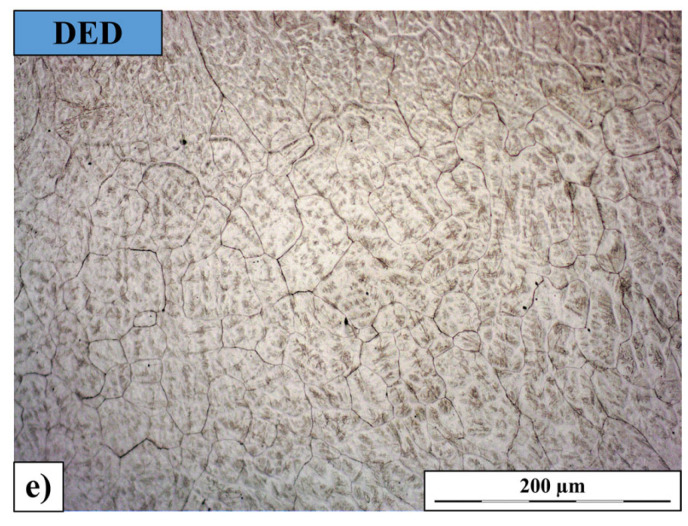
Microstructure of H13 steel etched with Nital after the printing process using: (**a**) FDMS (Desktop Metal Studio System), (**b**) FDMS (Zetamix), (**c**) Binder Jetting, (**d**) LPBF, (**e**) DED.

As illustrated in Figure 2, the observed microstructural differences reflect the fundamentally distinct metallurgical routes of the investigated AM processes. FDMS and Binder Jetting are sinter-based technologies, whereas LPBF and DED rely on full melting and resolidification, each imposing a characteristic thermal history and cooling rate that promotes non-equilibrium microstructures with possible segregation, retained phases, and other solidification- or sintering-related features.

The microstructure of H13 steel after various printing processes in the etched state is shown in Figure 1. The microstructure of the sample printed using the FDMS method (Desktop Metal Studio System) (Figure 2a) contains numerous porous regions of varying sizes. Most grains in this sample have diameters exceeding 100 µm. Cracks occur along grain boundaries, particularly at the so-called triple junctions, where three grains meet. Etching reveals the presence of a martensitic structure with indistinct outlines. Some areas remain unetched despite prolonged etching time. A similar microstructure was observed for the sample produced by the Binder Jetting method (Figure 2c), though in this case, the number of pores and cracks is noticeably smaller.

In contrast, the sample produced using the FDMS method with the Zetamix system (Figure 2b) exhibits a different microstructure, where grains of similar size are filled with clearly etched needles of plate martensite surrounded by numerous fine precipitates of secondary phases. Along the grain boundaries, in many areas, fine lamellar pearlite began to crystallize. These boundaries, similar to those observed in the previous two methods, contain numerous cracks and pores.

A completely different microstructural pattern is visible in the sample printed using the LPBF method (Figure 2d). Grains with the fan-shaped morphology typical of this method reach lengths not exceeding 200 µm and heights below 100 µm. The grains are uniformly filled with packets of lath martensite. No cracks are observed along the grain boundaries, and the structure contains very few pores.

The microstructure of the sample produced using the DED method with the Meltio system (Figure 2e) is less homogeneous. It contains regions with equiaxed grains smaller than 50 µm, as well as columnar grains less than 50 µm thick but over 200 µm long. This is a typical dendritic structure characteristic of this manufacturing method. A dendritic structure is also clearly visible in the sample produced by the LPBF method. The structure contains very few pores, though the grain boundaries appear thicker compared to the LPBF sample. Regardless of the region, the grains are filled with both plate and lath martensite.

### 3.2. Porosity of Printed Parts

**Figure 3:**
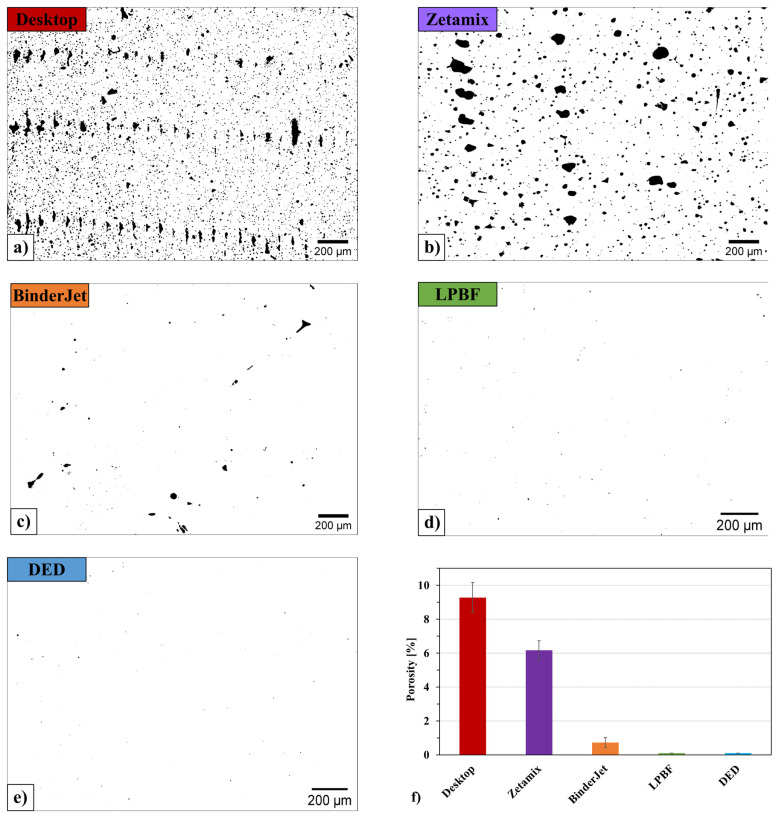
Microstructure of unetched H13 steel, including only pores with an area of at least 10 µm^2^, after the printing process using: (**a**) FDMS (Desktop Metal Studio System), (**b**) FDMS (Zetamix), (**c**) Binder Jetting, (**d**) LPBF, (**e**) DED; (**f**) comparison of porosity for different methods.

The analysis of the microstructure of unetched samples made it possible to assess the porosity level of H13 steel depending on the applied 3D printing method (Figure 3). The studies showed that the samples produced by the FDMS methods exhibited the highest porosity (Figure 3a,b).

**Figure 4:**
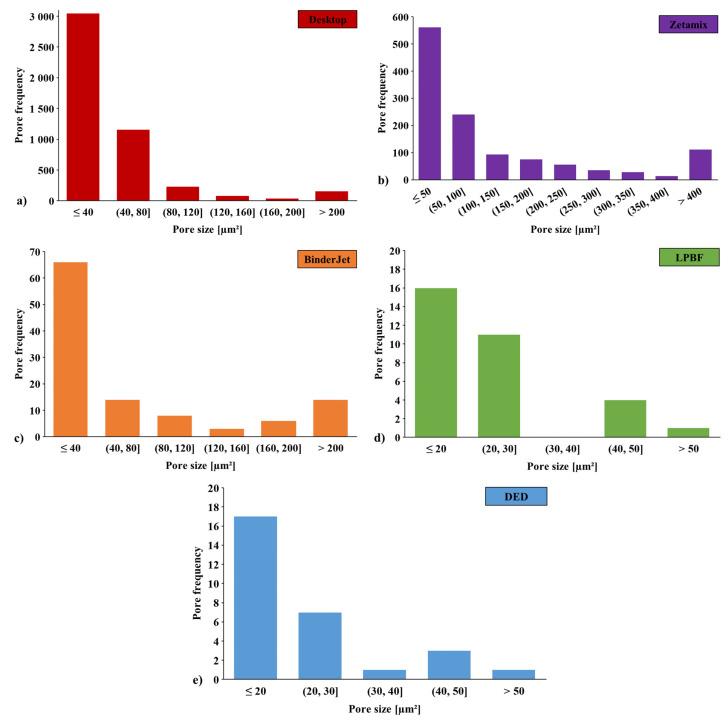
Pore distribution histogram of H13 steel, including only pores with an area of at least 10 µm^2^, after the printing process using: (**a**) FDMS (Desktop Metal Studio System), (**b**) FDMS (Zetamix), (**c**) Binder Jetting, (**d**) LPBF, (**e**) DED.

The pores observed in samples manufactured using both of these methods can be divided into two groups. The first group consists of large pores uniformly distributed between individual tracks. The second group includes small pores randomly distributed both within the grains and along their boundaries. To clarify and discuss the porosity, Figure 4 presents the pore distribution histogram for each AM method.

For the sample produced using the Binder Jetting method (Figure 3c), a significantly smaller number of pores was observed. Most of them are located between the individual grains and tracks. In the samples produced using the LPBF and DED methods (Figure 3d,e), only small, randomly distributed pores of very low size are present.

Quantitative analysis (Figure 3f) of pore content revealed that the porosity of samples produced by FDMS was 9.3% and 6%, respectively. The porosity of the sample produced using the Binder Jetting method was 0.7%, while for the LPBF and DED methods, it did not exceed 0.1%.

Due to the high porosity of the FDMS samples (6.0–9.3%), which significantly exceeded the 0.1–0.7% observed in other samples, both FDMS methods were excluded from further analysis. Such extensive internal defects, like pores, would have led to large variations in hardness measurements and compromised the reliability of microstructural evaluation. Additionally, the porous structure increased the risk of cracking during quenching and could promote local decarburization. Consequently, FDMS samples could not be reliably compared to higher-density materials, and their detailed discussion is therefore omitted from Section 3.3 and Section 3.4.

### 3.3. Evaluation of Heat Treatment Influence on Hardness of Printed H13 Steel

**Figure 5:**
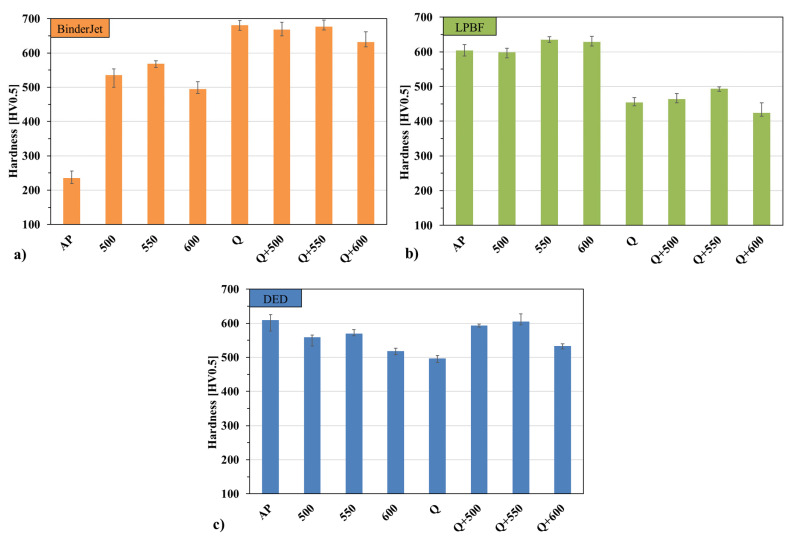
Hardness of H13 steel for different heat treatment variants after the printing process using: (**a**) Binder Jetting, (**b**) LPBF, and (**c**) DED.

One of the key parameters of hot-work tool steel is its high hardness, which determines, among other things, its resistance to abrasive wear. To achieve the required hardness of the tested materials and ensure uniformity throughout their volume, the samples were subjected to different variants of heat treatment (quenching and tempering). The obtained hardness distributions after individual heat treatment variants for different printing methods are shown in Figure 5. Differences in the hardness distributions of additively manufactured samples result directly from the way heat treatment modifies the microstructure formed immediately after the printing process.

Directly after printing, the lowest hardness was observed in the material produced by the BJ method, with a value of approximately 235 HV0.5 (Figure 5a). For the LPBF (Figure 5b) and DED (Figure 5c) methods, these values were significantly higher—around 600 HV0.5. After heat treatment, the hardness of the BJ-printed material increased significantly to about 500 HV0.5—this increase was already observed after tempering alone at 550 °C. After quenching, depending on the tempering temperature, hardness ranged between 620 and 680 HV0.5.

In the case of samples produced using LPBF and DED methods, the material immediately after printing already exhibited high hardness, about 600 HV0.5 in both cases. After tempering conducted directly after printing, the hardness of the LPBF material slightly increased, reaching about 620 HV0.5 at 550 °C, whereas for DED, a slight decrease was observed after tempering. After full heat treatment (quenching + tempering), the DED material showed an increase in hardness to around 600 HV0.5—a value similar to that obtained directly after printing. Regardless of the applied manufacturing method, tempering at 550 °C resulted in the highest hardness increase among the tempered samples in each group, indicating the existence of a favorable range of tempering parameters for the investigated materials.

### 3.4. Results of Microstructure Analysis After Heat Treatment Processes

A comprehensive analysis of the material’s microstructure was carried out for the as-printed condition and after various heat treatments, including tempering alone as well as quenching followed by tempering. These analyses were performed for three technologies (BJ, LPBF, and DED). For these, a comparison of the microstructures was made for the conditions: tempering at 550 °C, quenching, and quenching & tempering at 550 °C.

**Figure 6:**
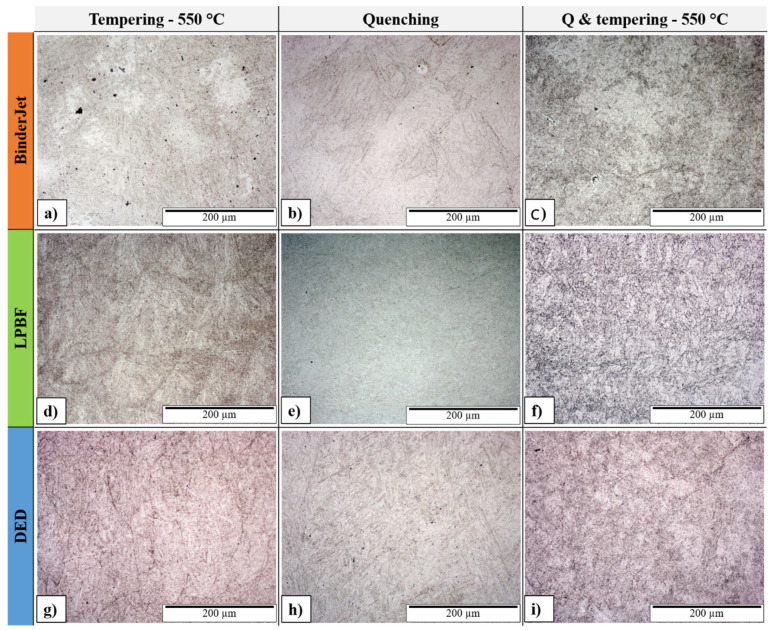
Microstructure of H13 steel etched with Nital after various heat treatment processes for samples printed using the Binder Jetting, LPBF, and DED methods.

The microstructure of the H13 steel sample printed using the Binder Jetting process in the tempered condition at 550 °C (Figure 6a) is rich in post-martensitic structures, which were difficult to reveal in the as-printed sample. The boundaries between the tempered martensite grains contain fine precipitates formed during the tempering process. Quenching of the sample (Figure 6b) did not eliminate the original grain boundaries (boundaries between tracks); however, a new structure appeared, characterized by clearly visible martensite plates inclined to one another at a characteristic angle of about 60°. The boundaries between the original grains became much less distinct. After tempering the quenched sample at 550 °C (Figure 6c), the presence of fine needle-like martensite and small carbide precipitates can be observed.

The microstructure of the H13 steel sample printed using the LPBF process and tempered at 550 °C (Figure 6d) is very similar to that of the as-printed sample. The texture resulting from the printing process has been preserved; however, individual packets of lath martensite are much more distinct due to the presence of fine precipitates along their boundaries and interdendritic regions. Quenching (Figure 6e) led to the disappearance of the original texture formed during printing. The structure became homogeneous, difficult to etch, and rich in fine needle-like martensite. A similar microstructure can be observed after tempering this sample at 550 °C (Figure 6f). The martensite grains are much more distinct, again due to the presence of fine carbide precipitates.

The microstructure of the H13 steel sample printed using the DED (Wire LMD) process and tempered at 550 °C (Figure 6g) is rich in martensite plates present within the primary grains formed during printing. As in the previous samples, stronger etching occurs due to the presence of fine precipitates along the grain boundaries. Unlike the sample printed by the SLM method, quenching of the DED-printed sample did not lead to the disappearance of its original texture (Figure 6h). The microstructure of this sample is clearly rich in martensite plates inclined to one another at the characteristic angle of about 60°. These plates become more distinct after tempering at 550 °C (Figure 6i), accompanied again by the presence of fine precipitates typical of tempered steels.

## 4. Discussion

Samples produced by the FDMS (Desktop Metal Studio System and Zetamix) and Binder Jetting methods exhibited the highest porosity. As observed in many areas, pores and cracks were present at the junctions of multiple grains and at the interfaces between deposition tracks and layers. The grain size for these three methods was very similar, as was their shape (approximately equiaxed). The process of forming bulk samples through powder sintering also promotes the formation of voids and cracks along their boundaries. The metal sintering process cannot completely fill these voids, which have specific shapes resulting from the stacking of successive material layers. This behavior is consistent with the microstructure evolution during supersolidus liquid phase sintering (SLPS) of binder jet-manufactured H13, where inter-particle boundaries serve as preferential sites for void formation and carbide stringers decoration [45].

A different situation is observed for the LPBF and DED methods, where, regardless of the track size, its flattened semicircular (“fan-like”) shape plays an important role. This shape, which also results from the spreading of molten metal on the surface of the sample, ensures the formation of a bulk material free from large pores. These technologies also guarantee sufficient metal fluidity to fill potential voids, which is achieved through the appropriate process parameters [46].

**Figure 7:**
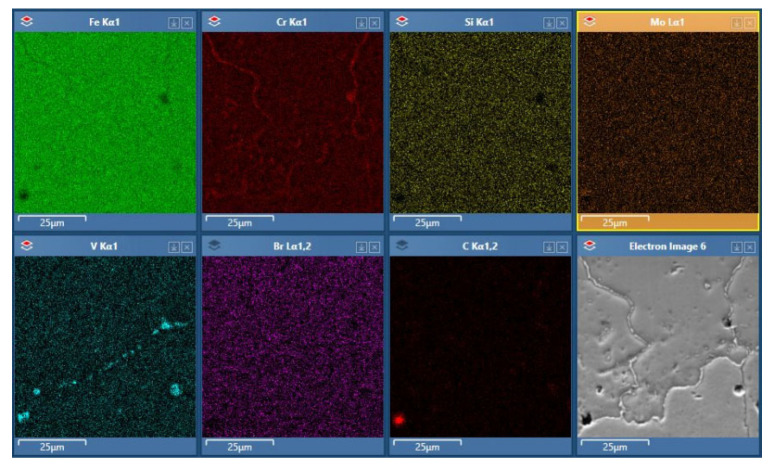
Elemental distribution in H13 steel printed using the Binder Jetting method after EDS analysis.

The hardness of the material printed using the Binder Jetting method results from the temperature–time profile of the sintering process, which determines the material properties. The material was likely partially hardened, as a significant increase in hardness was observed after tempering. Such a considerable increase in hardness was possible, among other reasons, because after the printing and sintering processes, the material contained many regions with a non-uniform chemical composition. This is confirmed by EDS analysis (Figure 7) of selected areas of the material, which clearly shows the segregation of certain elements (vanadium and chromium) in specific circular regions visible in the microstructure images. Dendritic segregation in H13, particularly of vanadium and chromium, is a well-documented phenomenon during powder metallurgical processes and solidification. During subsequent tempering, diffusion of atoms from regions of high concentration leads to precipitation of secondary carbides (M_7_C_3_, M_23_C_6_, VC) from these segregated zones, a process known as secondary hardening [47].

These areas were also identified in materials printed using the FDMS methods (Desktop Metal Studio System and Zetamix). During tempering, diffusion of atoms occurred from regions of high concentration, leading to the formation of new strengthening precipitates. The precipitation of molybdenum- and vanadium-rich carbides during tempering is responsible for the secondary hardening phenomenon, whereby hardness increases at intermediate tempering temperatures (typically 500–650 °C for H13) due to the replacement of coarse iron carbides by finely dispersed alloy carbides. Subsequent quenching resulted in a significant increase in the hardness of the tested steel to approximately 700 HV. The hardness was so high that it could not be further increased by additional tempering processes [48].

In the case of H13 steel samples printed using the LPBF method, crystallization of the material initially occurs at very high temperatures. The crystallized material is then subjected to subsequent thermal cycles resulting from the deposition of additional layers of material. Depending on the process parameters, the first thermal cycles cause material recrystallization (austenitization temperature), while the following cycles lead to its tempering. Repeated thermal cycling during LPBF induces γ−α′−γ transformations, generating and progressing misorientations within austenite grains, resulting in partial recrystallization of the initially solidified austenite. As a result of these repeated thermal cycles, the inner regions of the printed sample are effectively tempered steel. However, due to the small amount of material deposited in each track, the tempering process is brief and not fully completed. Therefore, performing additional tempering led to an increase in the hardness of H13 steel, as the process could then proceed to completion, resulting in a secondary hardening effect [49].

Subsequent quenching, as seen in the microstructure images, caused a complete transformation of the original structure. After quenching, the structure became relatively coarse-grained, which later had a positive influence during the tempering process. The prior austenite grain size after quenching plays a critical role in martensitic transformation and subsequent tempering response; larger prior austenite grains can accommodate higher densities of dislocations and facilitate carbide precipitation during tempering. To explain this effect more thoroughly, an analysis of the prior austenite grain size after quenching would be required [50].

A different situation was observed for the DED process, where a single deposition track is much larger than in the LPBF method. In this case, the large volume of newly deposited material led to complete tempering of the H13 steel during processing. The higher heat input and slower cooling rates characteristic of DED result in more extensive tempering of previously deposited layers, approaching equilibrium conditions. Consequently, additional tempering did not increase the material’s hardness, since the process had already been effectively completed. Further tempering caused additional diffusion of carbon atoms, leading to over-tempering of the martensitic structure and, as a result, its softening. Re-quenching and tempering of H13 steel restored the hardness to its original level [51,52].

## 5. Conclusions

The applied 3D printing methods can be summarized in terms of the resulting microstructure and properties, as well as other technological limitations such as part fabrication time, process costs, and the cost of the printing equipment. The cost aspect is relative and cannot be the main subject of analysis; however, some general assumptions were made that are independent of the evolving market conditions. Table 3 below presents a comparison of the applied H13 steel printing methods in the context of quality, cost-effectiveness, and process efficiency.

The cost data presented in the table are indicative and based on quotations collected during 2023–2024, when this study was conducted. They may serve as a helpful reference when selecting a suitable technology; however, we encourage readers to follow ongoing developments and technological progress. Nevertheless, for applications where lower density of printed parts is acceptable, the most attractive option appears to be FDMS printing using composite filaments filled with metal powders, mainly due to its accessibility.

Density and Porosity: LPBF achieved the highest density (99.9%), followed closely by DED (>99.9%) and Binder Jetting (99.3%). FDMS methods exhibited substantially lower densities, with Zetamix reaching 94% and Desktop Metal 90.7%, making these methods unsuitable for applications requiring near-theoretical density but acceptable for less demanding structural applications.

On the basis of the aforementioned data and comprehensive discussion of the results, the following conclusions have been derived:

1. Hardness After Heat Treatment: Binder Jetting, LPBF, and DED all demonstrated hardness development after heat treatment (marked as “+”), indicating successful response to the applied thermal cycles. FDMS methods showed no hardness development after heat treatment (“−”) because of high porosity which causes limited possible applications compared to other technologies.
2. Microstructure: LPBF and DED produced relatively uniform microstructures with controlled grain morphology. Binder Jetting exhibited segregation of alloying elements (vanadium, chromium) in specific regions, requiring diffusion during tempering to achieve secondary hardening. FDMS methods demonstrated more extensive segregation patterns due to inter-powder boundaries and limited material homogenization during sintering.
3. Production Cost and Equipment Accessibility: FDMS (particularly Zetamix) offers the lowest cost of printing devices and greatest accessibility, making it the most attractive for applications tolerating lower density. Binder Jetting requires one of the most expensive printing systems on the market, justified only by high precision requirements and substantial order volumes. LPBF and DED occupy intermediate equipment cost ranges, with competitive material costs of 60–80 EUR/kg and 25–40 EUR/kg, respectively, compared to 100–700 EUR/kg for Binder Jetting and FDMS methods.
4. Production Time and Process Efficiency: Binder Jetting demonstrated the highest deposition rate (5.5 kg/h apparent throughput), followed by DED (1–2 kg/h), FDMS methods (1.5–3 kg/h), and LPBF (0.1–0.5 kg/h). For applications requiring lower density, FDMS printing using composite filaments filled with metal powders is the most attractive option due to its accessibility and reasonable deposition efficiency. For applications requiring densities close to 100% and very high dimensional accuracy, LPBF performs best. For large parts where high surface precision is not essential, DED based on wire melting is a suitable choice. Binder Jetting represents a viable option for high-precision, small-series production only if large-scale orders are available to offset the high equipment cost. Ultimately, technology selection must account for part geometry, size constraints, required density, dimensional accuracy, production volume, and total cost of ownership.

## Figures and Tables

**Table 1:** Nominal Chemical Composition of AISI H13 Steel [42].

C	Si	Mn	Cr	Mo	V	P	S	Fe
0.32–0.45	0.80–1.20	0.20–0.60	4.75–5.50	1.10–1.75	0.80–1.20	≤0.030	≤0.030	Balance

**Table 2:** Materials characterization for AM Processing Routes.

AM Method	Feedstock Type	Morphology	Particle Size/Distribution	Source/Notes
**LPBF**	Gas-atomized powder	Spherical	Dv_10_ = 26.4 μm, Dv_50_ = 45.0 μm, Dv_90_ = 62.4 μm;	Commercial supplier
**FDMS Desktop Metal**	Powder in thermoplastic binder	Irregular (powder composite)	Dv_10_ = 11.5 μm, Dv_50_ = 34.3 ± 3.2 μm, Dv_90_ = 63 μm	Extracted from H13 filament
**FDMS Zetamix**	Loose powder from filament	Irregular	Dv_10_ = 9.5 μm, Dv_50_ = 18.4 ± 1.6 μm, Dv_90_ = 44 μm	Extracted from H13 filament
**Binder Jetting**	Gas-atomized powder	Spherical	Dv_10_ ≈ 10–15 μm, Dv_50_ ≈ 25 μm, Dv_90_ ≈ 45 μm [34]	Commercial supplier; optimized for binder infiltration
**DED**	Wire feedstock (solid)	Wire (1.0 mm diameter)	Diameter: 1.0 mm	H13 composition (AISI H13 equivalent)

Note: Dv_10_, Dv_50_, and Dv_90_ represent the particle diameter at 10%, 50%, and 90% cumulative volume distribution, respectively, as determined by laser diffraction analysis (ISO 13320).

**Table 3:** Comparative Summary of AM methods for printing of H13 steel.

Method	FDMS (Desktop Metal)	FDMS (Zetamix)	Binder Jetting	LPBF	DED
density [%]	90.7	94	99.3	99.9	>99.9
hardness as-printed	−		−	+	+
hardness after HT	−		+	+	+
deposition rate [kg/h]	2–3	1.5–2.5	5.5	0.1–0.5	1–2
accuracy of printed part	low	low	high	high	medium
cost of material [EUR/kg]	500–700	500–700	100–500	60–80	25–40
cost of printing device	medium	low	high	medium	medium

## Data Availability

The original contributions presented in this study are included in the article. Further inquiries can be directed to the corresponding author.
